# Uridine phosphorylase 1 associates to biological and clinical significance in thyroid carcinoma cell lines

**DOI:** 10.1111/jcmm.14612

**Published:** 2019-09-09

**Authors:** Yaoyao Guan, Adheesh Bhandari, Xiaohua Zhang, Ouchen Wang

**Affiliations:** ^1^ Department of Thyroid and Breast Surgery The First Affiliated Hospital of Wenzhou Medical University Wenzhou China

**Keywords:** epithelial‐mesenchymal transition (EMT), lymph node metastasis, metastasis, proliferation, thyroid carcinoma, UPP1

## Abstract

Thyroid cancer incidence has been continuity increasing worldwide. Uridine phosphorylase 1 (UPP1) is a protein‐coding gene and has been detected that UPP1 was the higher expression in many solid malignancies, just as head and neck cancers, breast cancer, compared with paired normal tissue. But the act of UPP1 in thyroid cancer is not explicit. In this article, we investigate the function of UPP1 expression in thyroid cancer. The Cancer Genome Atlas (TCGA) unpaired thyroid cancer and normal RNA‐seq data were downloaded, and our paired thyroid cancer and normal samples were analysed by a polymerase chain reaction. The expression of UPP1 was regulated by transfected small interfering RNA, and the function of UPP1 was determined via migration, invasion and cell proliferation assays. Western blot assay was achieved to determine the UPP1 expression correlates with the function of 5‐FU regulate epithelial‐mesenchymal transition. The significant upregulation of UPP1 in thyroid cancer tissues compared with normal thyroid tissues was revealed by our data and TCGA data. UPP1 overexpression was significantly correlated with lymph node metastasis, tumour stage and tumour size. In the cell, experiments showed that UPP1 low expression significantly suppressed the migration, invasion and proliferation. Western blot assay proves the effect of UPP1 expression on 5‐FU regulates epithelial‐mesenchymal transition pathway. UPP1 plays a crucial oncogene in thyroid cancer. Our findings indicate that UPP1 might be a biomarker of thyroid cancer and may act by regulating epithelial‐mesenchymal transition (EMT).

## 1 INTRODUCTION

Thyroid cancer has been continuously increasing and extraordinarily prevalent worldwide.^1^, ^2^ The US National Cancer Institute anticipated 53 990 new cases besides 2110 deaths numbers of patients due to thyroid cancer in the USA in 2018.^3^, ^4^ A recent SEER data analysis revealed the average increase in thyroid cancer mortality was 1.1% per year. With the extraordinary prevalence of neck ultrasonography in the last two decades, we have made development in initial diagnosis and surgical management. The occurrence and progression of thyroid carcinoma are generally decided by genomic variation, such as copy number variants, and single nucleotide polymorphisms lead to mutation of oncogenes and tumour suppressor genes. BRAF plays a crucial part in the activation of the MAPK pathway. The BRAFV600E mutation has been a significant biomarker for early diagnosis of thyroid cancer.^5^, ^6^ In 2013, TERT promoter mutations C228T and C250T were discovered, which represents another significant milestone in the field of thyroid cancer.^7^ A strong synergistic effect on the insistence of thyroid cancer happened in the co‐occurrence of TERT and BRAF V600E promoter mutations, which has been using for the early finding and optimal management of thyroid cancer. We have made progress in genomic mutations research, but the pathogenesis and many characters of thyroid cancer are still unclear. Hence, seeking for latest potential molecular markers and revealing molecular mechanisms in the occurrence and progression of thyroid carcinoma was essential.

Uridine phosphorylase catalyses the reversible phosphorolysis of uridine to uracil.^8^, ^9^ There is an article discovered uridine phosphorylase activity in tumours appeared approximately twofold to threefold higher associated with normal tissues.^10^ The high expression of Uridine phosphorylase in oral squamous cell carcinoma has been proved as a prognostic marker.^11^ Uridine phosphorylase has two isoforms in human, UPP1 and UPP2.^12^, ^13^ UPP1 is ubiquitously distributed and massively expressed in the human body. UPP1 is a dimeric enzyme, playing an essential role in pyrimidine salvage and regulation of uridine homeostasis.^14^ It plays a vital role in the stimulation of pyrimidine nucleoside analogs. For example, 5‐fluorouracil could be activated to produce lots of metabolites that exert anti‐cancer activity.^15^ There are some studies that explained the high expression of UPP1 in tumours for the selectivity of chemotherapeutic agents^8^ as we all know that the current mainly treatment of thyroid cancer is surgery. However, we analysed the dates download from TCGA, about patients with thyroid cancer who have never accepted chemotherapy before. We still found significantly different expression from tumours to normal tissues. We have revealed a significant involvement among UPP1 and clinical characterization. But the function of UPP1 is still unknown in thyroid cancer. In this article, we will clarify the expression and effect of UPP1 in thyroid cancer.

## 2 MATERIALS AND METHODS

### 2.1 Patients and samples

Papillary thyroid carcinoma tissues and paired adjacent normal tissues were collected from 24 surgery patients. The samples were instantly achieved at the time of initial surgery and were frozen in liquid nitrogen immediately after lesion resection then stored at −80 Celsius before RNA extraction. All tumour tissues were histologically reviewed by two pathologists, and the cases were retrospectively reviewed by two senior pathologists to confirm the histological diagnosis. Major inclusion criteria were as follows: (a) patients with pathologically confirmed thyroid cancer in the primary tumour and without any severe diseases in other organs; (b) patients that had received total/near‐total thyroidectomy and had not received any radiotherapy; and (c) patients with a negative history of any other malignant tumours. Major exclusion criteria were as follows: (a) patients with a positive history of other malignant tumours; (b) patients with severe diseases such as heart failure, stroke and chronic renal failure; and (c) patients with a history of ^131^I therapy. All samples were confirmed as papillary thyroid carcinoma by histological diagnosis. All patient‐derived information was recorded following the protocols approved by the ethical standards of the Ethics Committee of the First Affiliated Hospital of Wenzhou Medical University (approval no. 2012‐57), and the written informed consent was obtained from every patient.

### 2.2 RNA extraction and qRT‐PCR

According to the manufacturer's protocol (Thermo Fisher Scientific), RNA isolation was performed using TRIzol reagent, and the ReverTra Ace qPCR RT Kit (Toyobo) was used for Reverse transcription. Real‐time quantitative polymerase chain reaction (RT‐qPCR) was analysed by Thunderbird SYBR qPCR Mix (Toyobo) according to the manufacturer's recommendations on applied Biosystems 7500 (Thermo Fisher Scientific). Every sample was in triplicate. The relative expression of UPP1 was controlled by GADPH. The primer sequences for UPP1 were as follows: UPP1, 5′‐ CCAATGCGGATGATAGTG ‐3′ (forward) and 5′‐ AACATCTGTGCGGGAACT ‐3′ (reverse).

### 2.3 Cell lines and cell culture

Professor Ming‐Zhao Xing of Johns Hopkins University School of Medicine supports us two TC cell lines TPC‐1 and BCPAP. Above‐mentioned cell lines were all cultured in RPMI 1640 (Invitrogen; Thermo Fisher Scientific, Inc, USA) which was supplemented with 10% foetal bovine serum (FBS; Invitrogen; Thermo Fisher Scientific, Inc). All cells were cultured in a 37°C humidified incubator with 5% CO_2_.

### 2.4 RNA interference

Small interfering RNA (siRNA) for UPP1 and negative control siRNA were acquired from GenePharma (Shanghai, People's Republic of China) for the regulation of gene expression. All operations of transfection severely followed the manufacturer's instructions. The transfection reagent was used Lipofectamine® RNAiMAX. The transfection efficiency on cell lines was verified by RT‐PCR. Cells were plated in a 6‐well plate 24 hours before transfection (TPC 4 × 10^4^/well, BCPAP 8 × 10^4^/well). Cells were incubated 48 hours in the incubator with 37°C, 5% CO_2_ after transfection of RNA isolation. All assays were achieved in triplicate. The siRNA sequences were as follows:

siRNA1: 5′‐ GCUGAAAGUCACAAUGAUUTT‐3′ (forward) and 5′‐ AAUCAUUGUGACUUUCAGCTT‐3′ (reverse); siRNA2: 5′‐ CCCAGCCUUGUUUGGAGAUTT‐3′ (forward) and 5′‐ AUCUCCAAACAAGGCUGGGTT‐3′ (reverse); siRNA3: 5′‐ CCGCUAUGCCAUGUAUAAATT‐3′ (forward) and 5′‐ UUUAUACAUGGCAUAGCGGTT‐3′ (reverse).

### 2.5 Cell proliferation assay

Cell Counting Kit‐8 (CCK‐8; Beyotime, China) assay: transfected TPC1 (1.5 × 10^3^ cells) and BCPAP (1.5 × 10^3^ cells) cells were plated in 96‐well plates overnight. Then, CCK‐8 is added to each well according to the company's instruction. After 2 hours incubation, it is then measured the absorbance at 450 nm (OD‐450). All assays were performed in triplicate.

Colony formation assay: Transfected TPC1 (1.5 × 10^3^ cells) and BCPAP (1.5 × 10^3^ cells) cells were plated in 6‐well plates and maintained by RPMI‐1640 with 10% FBS in the incubator with 37°C, 5% CO_2_ for 7‐10 days, when mature colony formation fixed with 4% paraformaldehyde for 15 minutes and stained with 0.4% crystal violet solution for 15 minutes at room temperature. The pictures were taken by the digital camera. All experiments were achieved in triplicate.

### 2.6 Transwell assays

Transfected cells incubated in 6‐well plate were cultured with trypsin enzyme digesting technique. 600 µL of RPMI1640 comprising 10% FBS was further added to the bottom chamber. Cells (3 × 10^4^) were suspended in 300 µL serum‐free RPMI1640 and seeded into the upper chamber. Then incubated at 37°C for 20 hours, the cells that migrated from upper to bottom were fixed and stained for 30 minutes with 0.01% crystal violet solution in PBS. The pictures were captured by the camera under the microscope. All experiments were accomplished in triplicate. The migration chambers and invasion chambers were purchased from Corning, NY, USA.

### 2.7 Apoptosis assays

An Annexin V/propidium iodide (PI) apoptosis kit (Nanjing KeyGen Biotech. Co., Ltd., Nanjing, China) were used to determine the apoptotic rates of TPC and BCPAP according to the company's advice. The cells were collected, rinsed three times by PBS and suspended in 1× binding buffer (Beyotime Institute of Biotechnology, Haimen, China) at a concentration of 1 × 106 cells/mL. Cell suspensions of 300 µL were stained with 5 µL of Annexin V‐fluorescein isothiocyanate and 5 µL of PI at room temperature for 15 minutes in the darkroom prior to analysis by flow cytometry (BD FACS Aria; BD Biosciences, Franklin Lakes, NJ).

### 2.8 Western blot analysis and antibodies

Total protein lysates were separated by 10% of sodium dodecyl sulphate‐polyacrylamide gel electrophoresis (Bio‐Rad, Berkeley, CA, USA) and electrophoretically transferred to polyvinylidene difluoride membranes (Millipore, Billerica, MA, USA). The membranes were blotted with 5% non‐fat milk for in TBST 2 hours at room temperature, then, washed three times by TBST and probed with rabbit anti‐N‐cadherin (1:2000 dilution), vimentin (1:2000 dilution), UPP1 (1:1000 dilution) and mouse‐β‐actin (1:2000 dilution) according to the company's protocol overnight at 4˚C. The membranes were then incubated with the antimouse IgG or anti‐rabbit IgG as a secondary antibody for 1.5 hours at room temperature. Each antibody was acquired from Abcam (Cambridge, MA, USA).

### 2.9 Statistical analysis

Data were shown as the means ± standard error. All statistical analyses were done using SPSS 23.0 software (SPSS Inc, Chicago, IL, USA); Student's t test was used for analysing two group comparisons. Multiple group comparisons were investigated with one‐way ANOVA. The differences were considered to be statistically significant at *P* < .05.

## 3 RESULTS

### 3.1 UPP1 expression is upregulated in thyroid cancer tissues and cell lines

**Figure 1:**
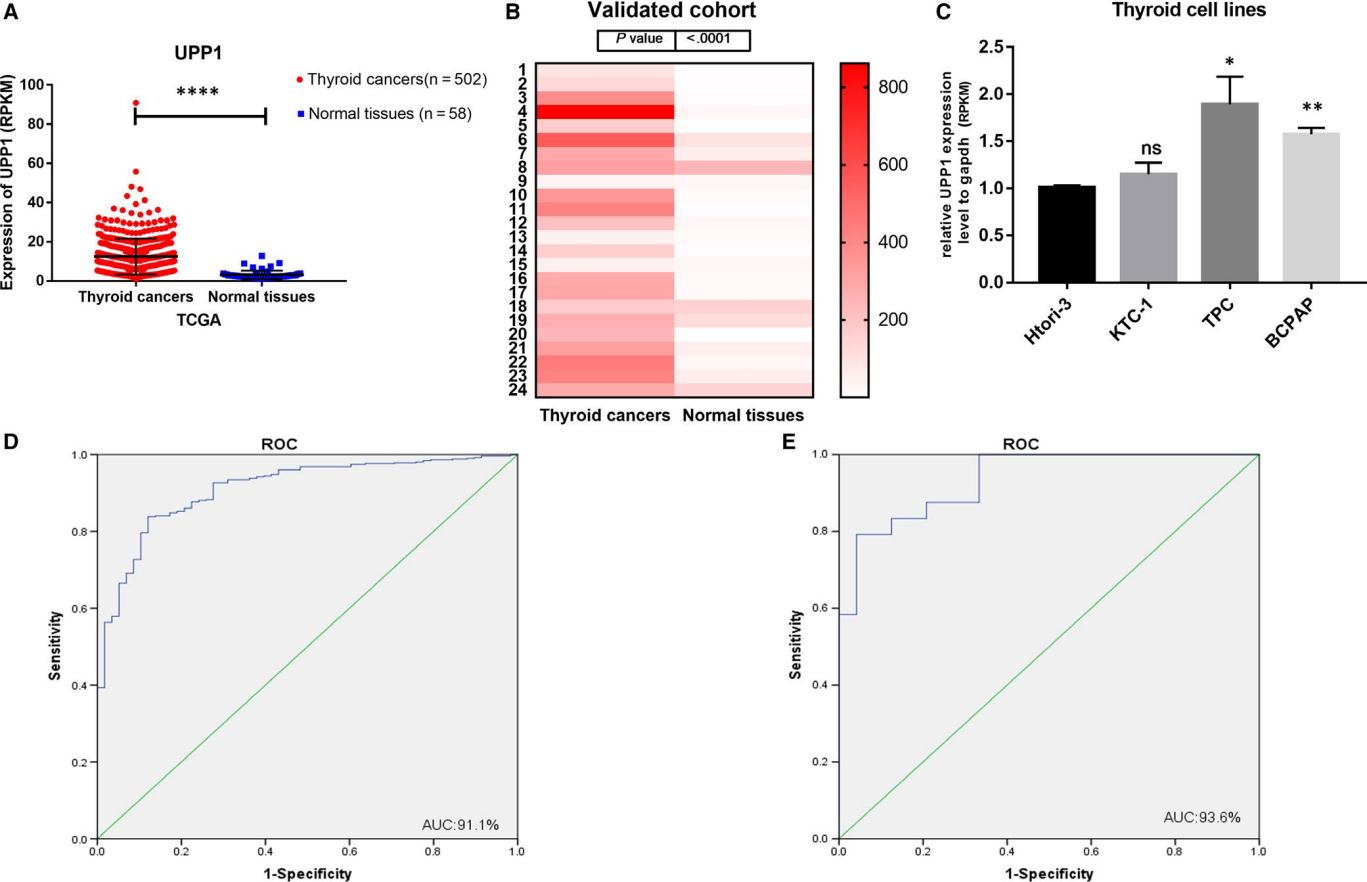
UPP1 expression in thyroid cancer in the TCGA cohort, validated cohort and thyroid cancer cell lines. A, The TCGA cohort contained 502 thyroid cancer tissues and 58 normal tissues. The analysis was done using unpaired t test. ****P < .0001. B, A hot map was used to describe the UPP1 expression examined by RT‐qPCR in 24 paired thyroid cancer tissues and adjacent noncancerous tissues (paired *t* test, *P* < .001). C, The relative expression of UPP1 gene using RT‐qPCR in thyroid cancer lines, UPP1 was expressed at a higher level in TPC and BCPAP. D, ROC curve for UPP1 expression to diagnose thyroid cancer in the TCGA cohort. The AUC was 91.1% (87.7%‐94.6%) (E) ROC curve for UPP1 expression to diagnose thyroid cancer in the validated cohort. The area under the ROC curve (AUC) was 93.6% (87.2%‐99.9%)

To explore the biological function of UPP1 in thyroid cancer. In our study, we assessed the UPP1 expression in the TCGA and measured the levels of UPP1 in thyroid cancer tissues and corresponding normal tissues by RT‐qPCR (24 pairs). We found UPP1 expression is significantly upregulated in thyroid cancer. TCGA (Thyroid cancers tissues 12.34 ± 0.4089, n = 502: Normal tissues 3.085 ± 0.2794, n = 58, unpaired t test (*P* < .0001) (Figure 1A). A heat map was made to observe the differential expression in collected surgical tissue samples (24 pairs thyroid cancer tissues and paired normal tissues, △d = −235 ± 185.7, paired *t* test, *P* < .0001). (Figure 1B). It suggests that UPP1 could be a potential marker to monitor thyroid cancer in a variety of thyroid cancer cell lines, and the expression of UPP1 also performs a higher level compared with normal cells line (compared with HTORI‐3, TPC, *P* < .05; BCPAP, *P* < .01) (Figure 1C).

### 3.2 UPP1 expression is associated with tumour size, tumour stage and lymph node metastatic

Analysed the clinical feature of the 502 thyroid cancer tissues from TCGA, patients were group by median value into low expression and high expression. We found the tumour stage (*P* = .018, <.05), tumour size (*P* < .001) and the lymph node metastasis (*P* < .001) were significantly different in higher expression group vs lower group (Table 1). But in our validated cohort, analysed the 24 thyroid cancer tissues, there is no statistical significance, tumour stage (*P* = .675), tumour size (*P* = .164) and the lymph node metastasis (*P* = .64), because the number of samples is too small to yield reliable results (Table 2).

**Table 1:** The relationship between UPP1 and clinicopathologic characteristics in the TCGA cohort

Clinicopathologic characteristics		High expression (%)	Low expression (%)	*P*‐value
Age	≤60	239	111	.131
	>60	61	38	
Gender	Female	86	37	.229
	Male	214	112	
Stage	I/II	184	107	.018*
	III/IV	116	42	
Tumour	T1‐2	160	110	<.001*
	T3‐4	140	39	
Lymph node	N0	124	104	<.001*
	Nx	176	45	
Status	Alive	272	136	.492
	Dead	28	13	

* *P* < .05.

**Table 2:** The relationship between UPP1 and clinicopathologic characteristics in the validated cohort

Clinicopathologic characteristics		High expression (%)	Low expression (%)	*P*‐value
Age	≤45	5	5	.26
	>45	10	4	
Gender	Female	8	4	.5
	Male	7	5	
Stage	I/II	10	6	.675
	III/IV	5	3	
Tumour	T1‐2	4	5	.164
	T3‐4	11	4	
Lymph node	N0	12	7	.64
	Nx	3	2	

### 3.3 Upregulation of UPP1 increased lymph node metastasis risk

To examine whether the expression level of UPP1 is correlated with lymph node metastasis, we investigated the connection between them by logistic regression. (Table 3) Univariate logistic regression investigation specified that the significant variables for lymph node metastasis were UPP1 expression (odds ratio [OR] 3.28, 95% CI 2.159‐4.985, *P* < .001), clinical stage (OR 3.555, 95% CI 2.356‐5.366, *P* < .001), gender (OR 0.653, 95% CI 0.430‐0.992, *P* = .046) and tumour size (OR 2.672, 95% CI 1.809‐3.947, *P* < .001), (Table 4). Multivariate logistic analysis revealed that UPP1 expression (OR = 3.053, 95% CI 1.957‐4.762, *P* < .001), clinical stage (OR = 5.117, 95% CI 3.110‐8.419, *P* < .001) and age (OR = 0.328, 95% CI 0.185‐0.582, *P* < .001) were associated with lymph node metastasis. That is to say, UPP1 expression serves as a risk of lymph node metastasis.

**Table 3:** Univariate logistic regression analysis for the lymph node metastatic risk

Clinicopathologic features	OR	95% CI	*P*‐value
UPP1 expression	3.28	2.159‐4.985	<.001*
Stage	3.555	2.356‐5.366	<.001*
Age	0.668	0.425‐1.049	.079
Gender	0.653	0.430‐0.992	.046*
Tumour size	2.672	1.809‐3.947	<.001*

* *P* < .05.

**Table 4:** Multivariate logistic regression analysis for the lymph node metastatic risk

Clinicopathologic features	OR	95% CI	*P*‐value
UPP1 expression	3.053	1.957‐4.762	<.001*
Stage	5.117	3.110‐8.419	<.001*
Age	0.328	0.185‐0.582	<.001*

* *P* < .05.

### 3.4 Upregulation of UPP1 increased Tumour size

There is a significant difference in tumour size between higher UPP1 expression group and lower UPP1 expression group in TCGA (Table 1). To validate the correlation of UPP1 expression with tumour size, we analysed by logistic regression (Table 5). Univariate logistic regression analysis indicated that the significant variables for tumour size were UPP1 expression (OR = 2.468, 95% CI 1.605‐3.794, *P* < .001), clinical stage (OR 9.486, 95% CI 6.067‐14.830, *P* < .001), age (OR 1.943, 95% CI 1.238‐3.049, *P* = .004), gender (OR 0.551, 95% CI 0.362‐0.838, *P* = .005) and lymph node metastatic (OR 2.672, 95% CI 1.809‐3.947, *P* < .001). (Table 6) Multivariate logistic analysis revealed that UPP1 expression (OR 2.398, 95% CI 1.467‐3.922, *P* < .001), clinical stage (OR 9.227, 95% CI 5.837‐14.586, *P* < .001) and clinical stage (OR 0.622, 95% CI 0.38‐1.017 *P* = .058) were associated with tumour size. All the above suggested the higher expression levels of UPP1 act as a risk for tumour growth in thyroid cancer.

**Table 5:** Univariate logistic regression analysis for tumour size risk

Clinicopathologic features	OR	95% CI	*P*‐value
UPP1 expression	2.468	1.605‐3.794	<.001*
Stage	9.486	6.067‐14.830	<.001*
Age	1.943	1.238‐3.049	.004*
Gender	0.551	0.362‐0.838	.005*
Lymph node metastatic	2.672	1.809‐3.947	<.001*

* *P* < .05.

**Table 6:** Multivariate logistic regression analysis for tumour size risk

Clinicopathologic features	OR	95% CI	*P*‐value
UPP1 expression	2.398	1.467‐3.922	<.001*
Stage	9.227	5.837‐14.586	<.001*
Gender	0.622	0.380‐1.017	.058

* *P* < .05.

### 3.5 UPP1 is a critical diagnostic, prognostic and predictive biomarker in thyroid cancer

The ROC curves for UPP1 expression to diagnose thyroid cancer in the TCGA and validated cohort both are >0.9 (AUC was 91.1% (87.7%‐94.6%) in TCGA and 93.6% (87.2%‐99.9%) in validated cohort), which usually indicate high accuracy. In this review, clinicopathologic characteristics associated with higher UPP1 expression in thyroid cancer has been discussed. In conclusion, UPP1 gene could be a critical diagnostic, prognostic and predictive biomarker in thyroid cancer.

### 3.6 Down‐regulation of UPP1 inhibited proliferation in thyroid cancer cell lines

**Figure 2:**
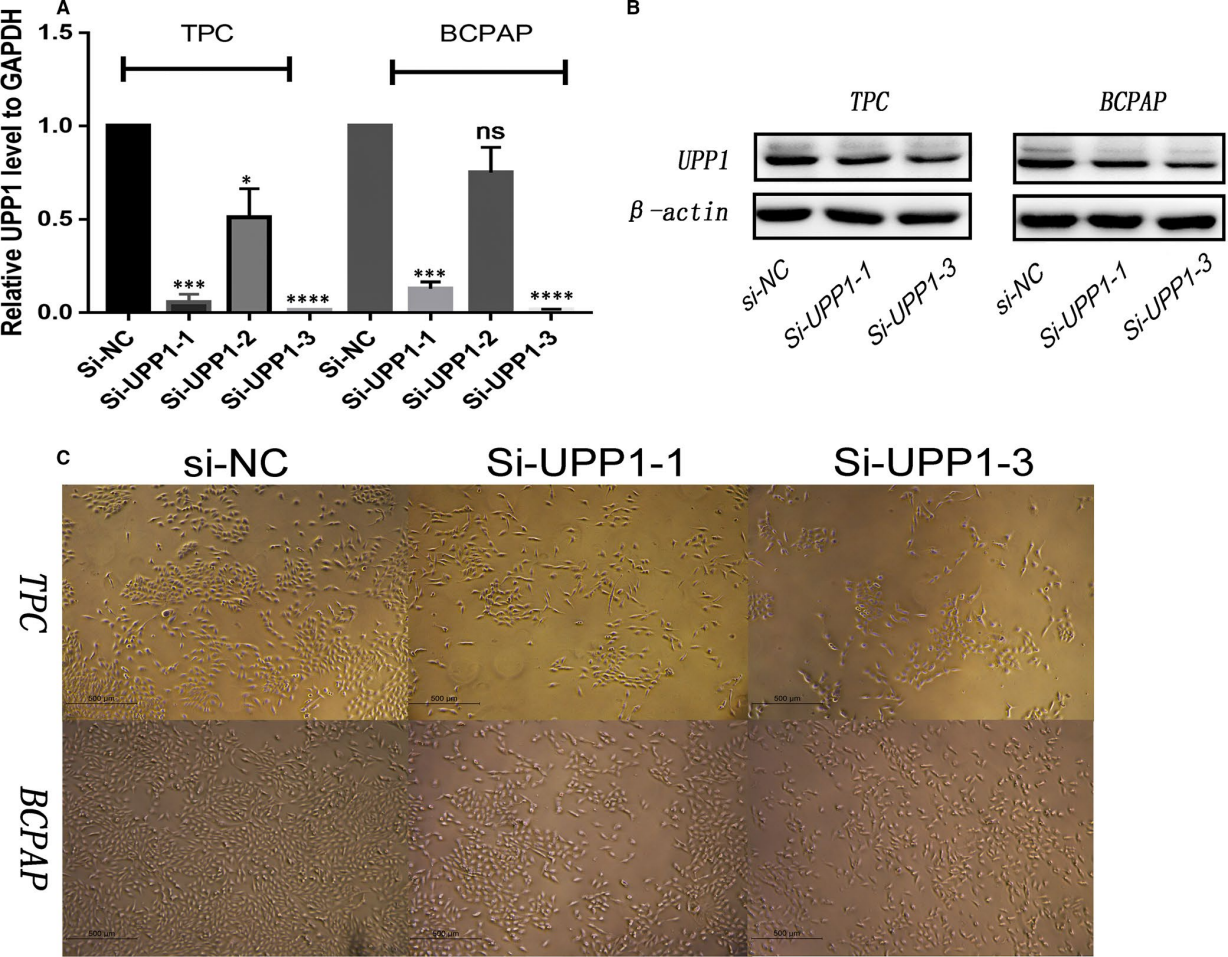
Down‐regulated UPP1 gene expression in thyroid carcinoma cells. A, TPC and BCPAP cells were transfected with siRNA‐NC, si‐UPP1‐1, si‐UPP1‐2 and si‐UPP1‐3 for 48 h and then analyse by PCR to revealed mRNA levels of UPP1 expression in these lines. Compared with corresponding control group, the knock‐down of UPP1 gene in si‐UPP1‐1 and the si‐UPP1‐3 group was effective. B, TPC and BCPAP cells were transfected with si‐UPP1‐1 or si‐UPP1‐3 or si‐NC for 48 h and protein levels of UPP1 are significantly reduced. C, Observed the cytomorphosis after knock‐down UPP1 under a microscope

**Figure 3:**
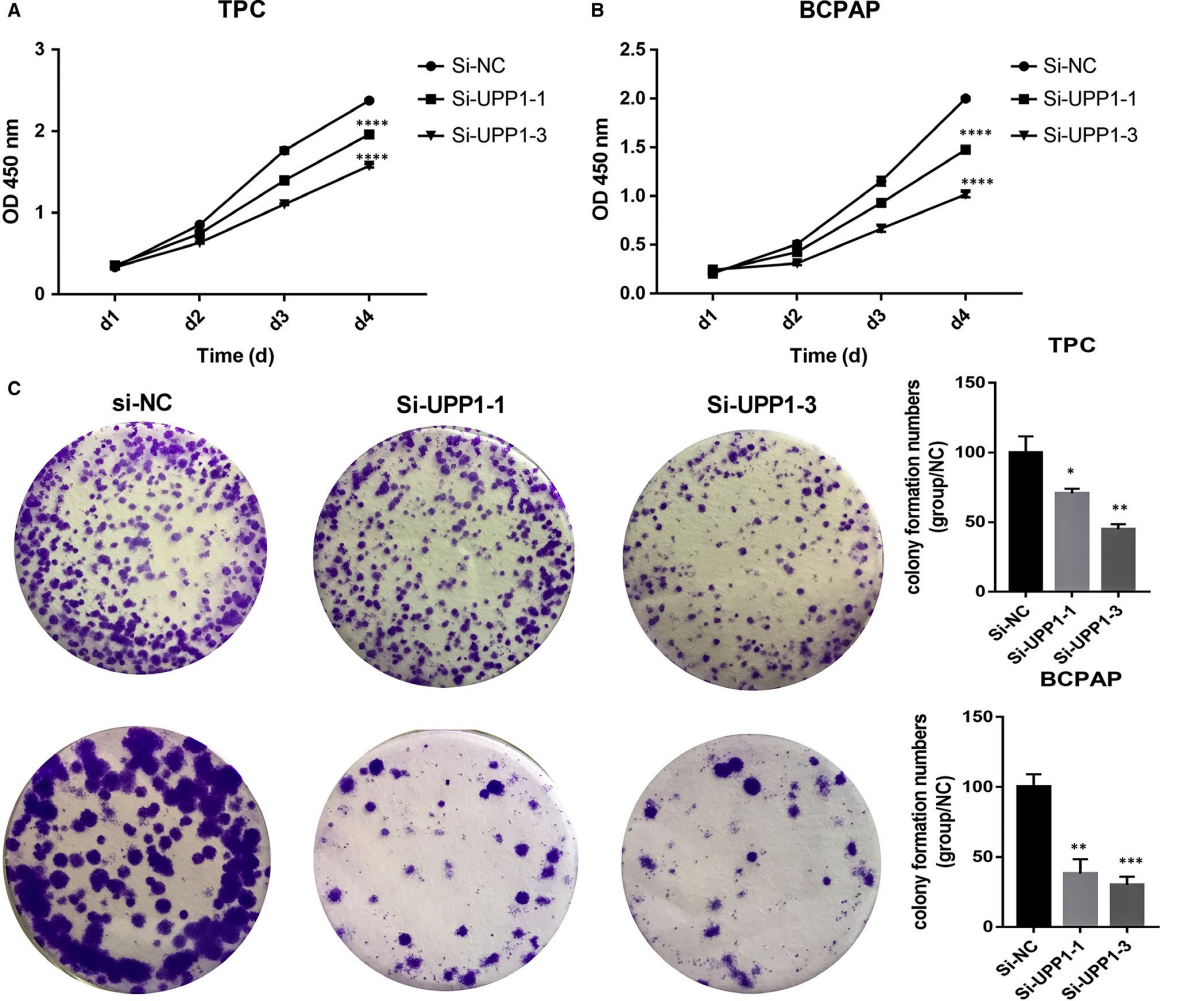
Down‐regulated UPP1 expression in thyroid carcinoma cells inhibited proliferation and colony formation. Cell proliferation assay: knock‐down of UPP1 in TPC (A) and BCPAP (B) cells compared with the corresponding control group, cell proliferation was measured using CCK‐8. Colony formation assay: TPC and BCPAP cells (C) transfected were cultured in 6‐well plates for 10‐14 days. *P < .05; **P < .01; ***P < .001；****P < .0001

We use the small interference RNA (siRNA) to silenced UPP1 (Figure2). To compare with the control group, we choose two effective silence groups (Si‐UPP1‐1, Si‐UPP1‐3) to complete the following experiments. We assess the ability of cell growth by CCK‐8 proliferation assay (Figure 3). And colony‐forming assay (Figure 3), we found that down‐regulating the level of UPP1 expression significantly inhibits proliferation in thyroid cancer cell line (TPC and BCPAP).

### 3.7 Down‐regulation of UPP1 inhibited migration and invasion

**Figure 4:**
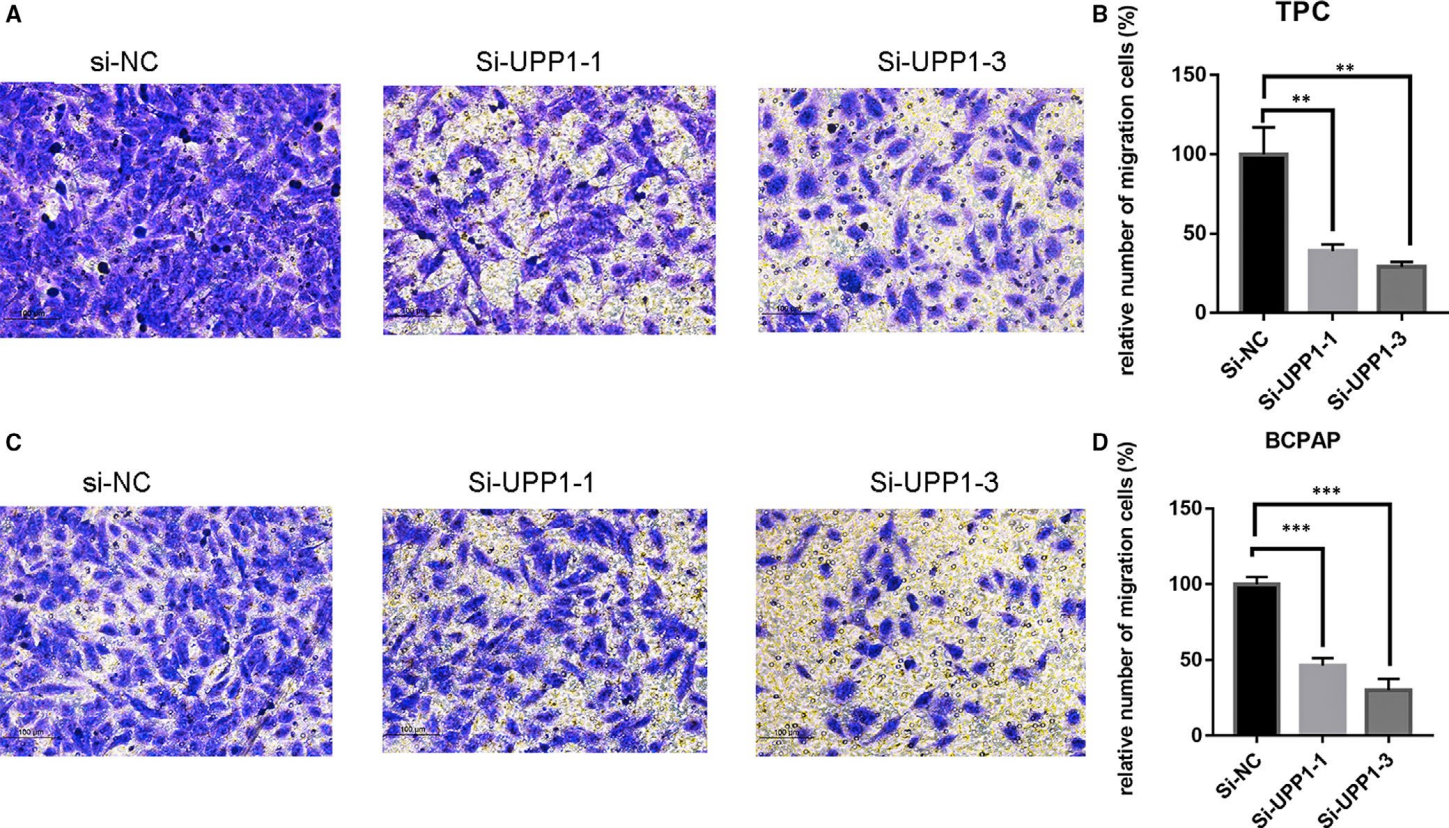
Down‐regulation of UPP1 gene expression in TPC and BCPAP cells inhibits migration. In TPC (A) and BCPAP (B)cells, transwell migration assays in down‐regulation UPP1 cells and their corresponding control cells. Quantitative results of migration assays. The stained cells were then counted manually from 3 randomly selected fields and normalized with cell proliferation. Comparison with the NC group using Student's *t* test. **P < .01; ***P < .001

**Figure 5:**
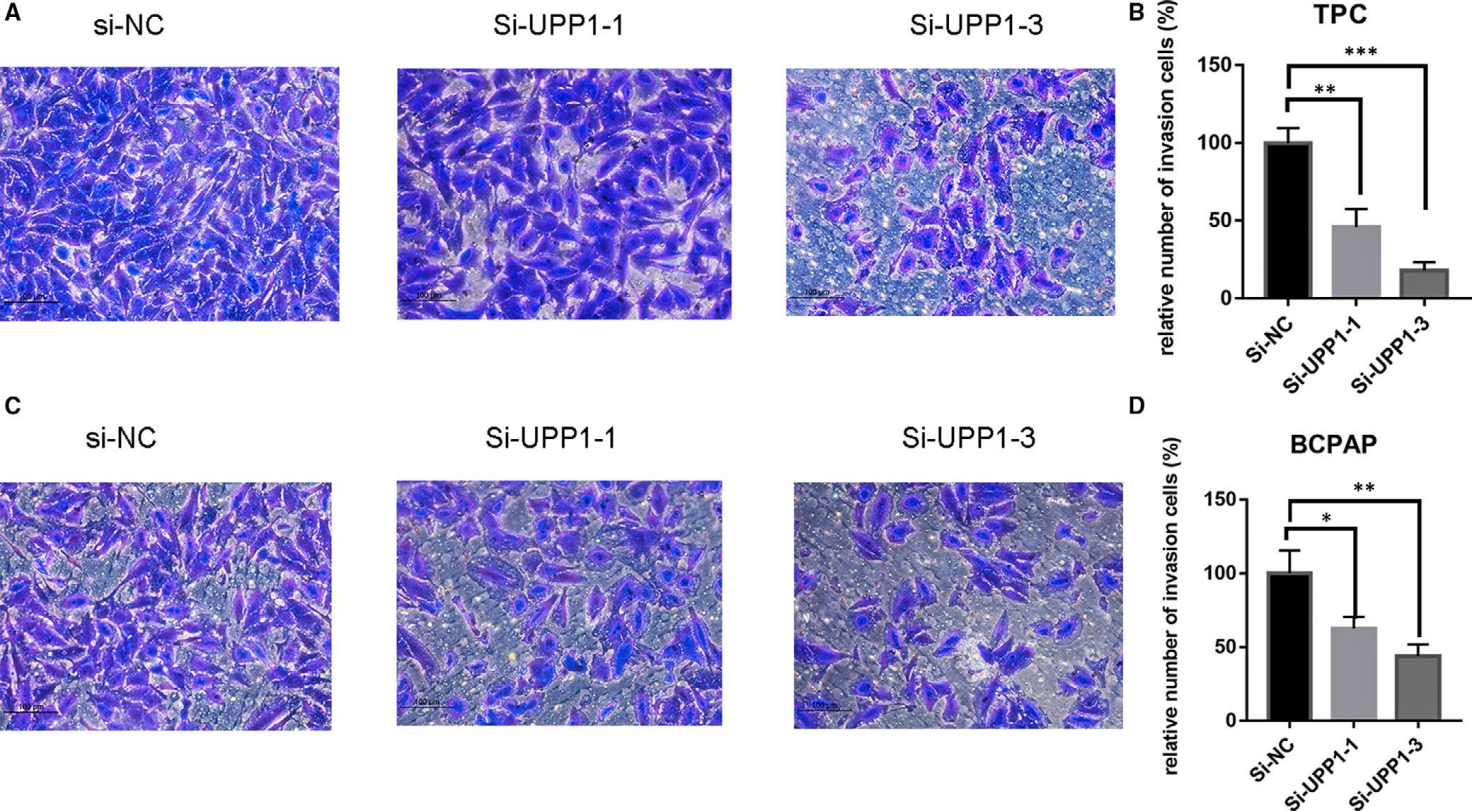
Down‐regulation of UPP1 gene expression in TPC and BCPAP cells inhibits invasion. In TPC (A) and BCPAP (B) cells, transwell invasion assays in down‐regulation UPP1 cells and their corresponding control cells. Quantitative results of invasion assays. The stained cells were then counted manually from 3 randomly selected fields. Comparison with the NC group performed with Student's *t* test. *P < .05; **P < .01; ***P < .001

Invasion and migration assay often use to evaluate tumour metastasis capacity. We found that down‐regulating the expression level of UPP1 inhibited the TPC and BCPAP migrate (Figure 4) and invade (Figure 5). From the upper chamber to the bottom chamber, compared with the control group. In a word, there is a positive correlation between the UPP1 expression and thyroid cancer metastasis capacity.

### 3.8 Knock‐down UPP1 induces the cell apoptosis of thyroid cancer cell lines in vitro

**Figure 6:**
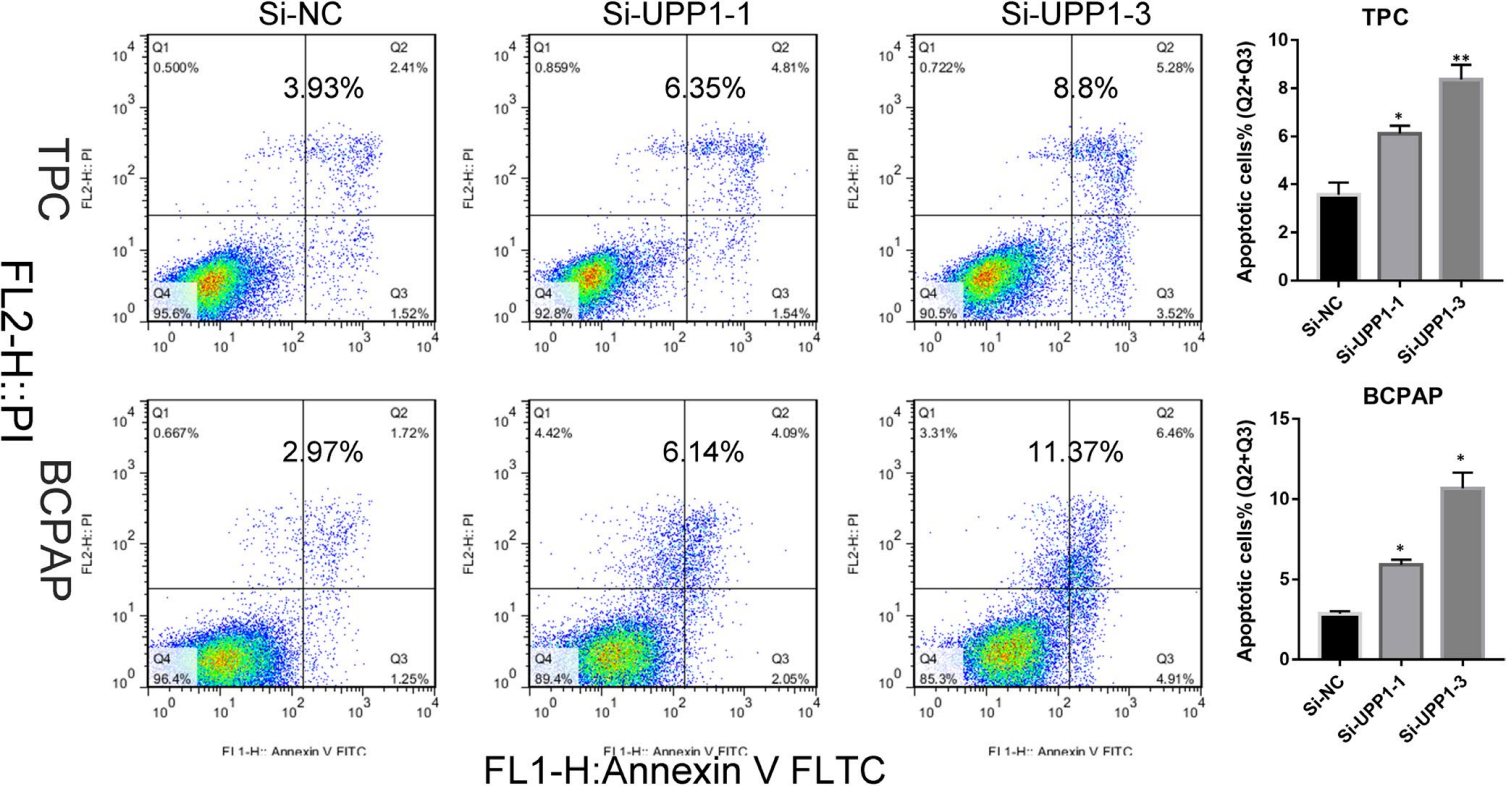
UPP1 knock‐down induces apoptosis in thyroid cancer cells. Apoptosis assay: Knocking down UPP1 in TPC and BCPAP cell lines were evaluated for apoptosis by Annexin V/PI. And the columns represent the mean of death cell numbers from at two independent experiments

We presumed that UPP1 also plays a role in the cycle of the cell, so flow cytometry was used to investigate the proportion of apoptotic cells transfected with si‐UPP1 cell lines. The results showed that knock‐down of UPP1 induced increased apoptosis in thyroid cancer cells (TPC, BCPAP), especially late‐stage apoptotic cells, compared with corresponding Si‐NC cell lines (Figure 6).

### 3.9 UPP1 promoted thyroid cancer migration and invasion by regulating EMT

**Figure 7:**
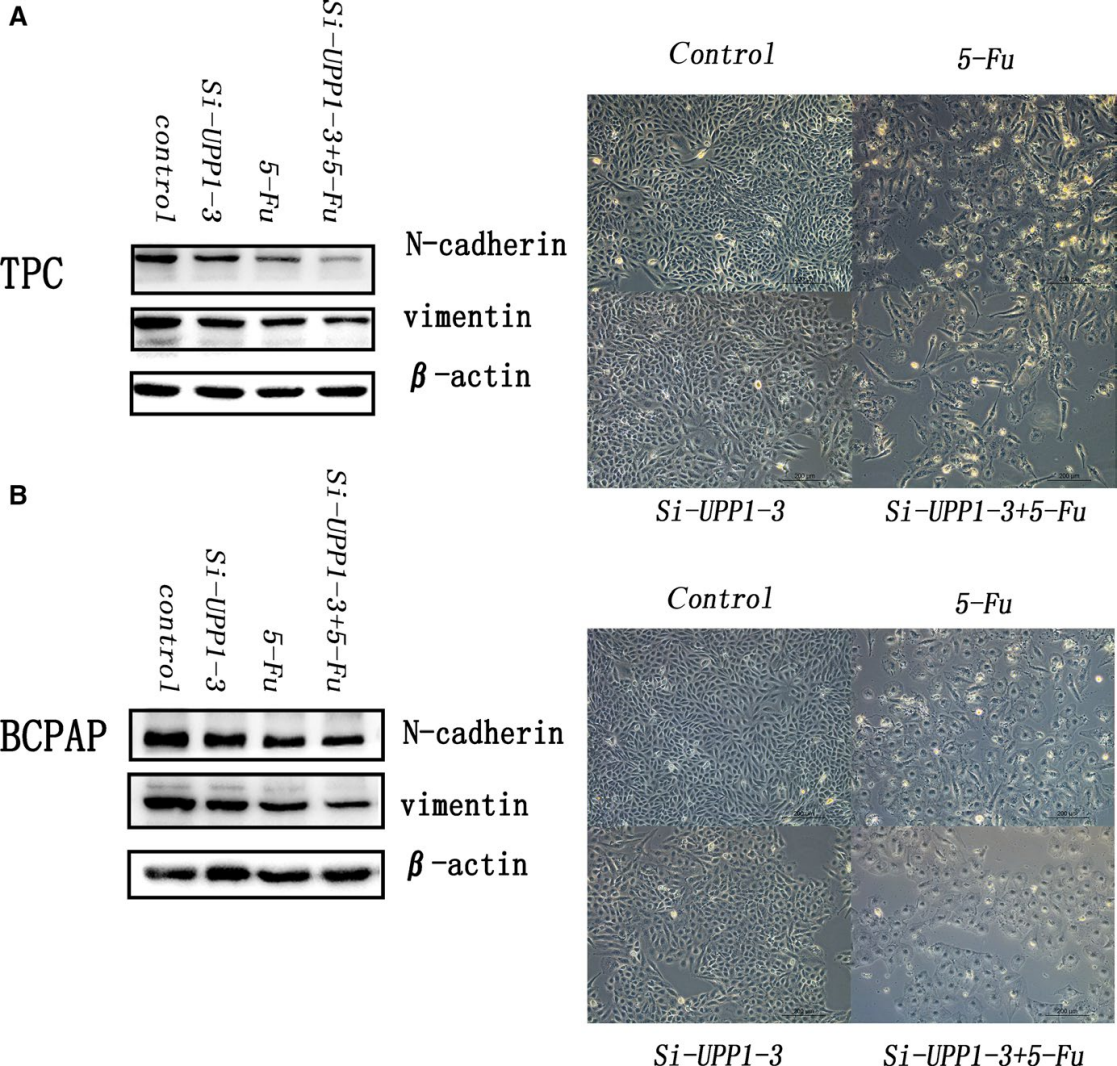
Effect of UPP1 with 5‐fu treatment or no treatment thyroid cancer cell lines (TPC and BCPAP) on epithelial‐mesenchymal transition proteins. N‐cadherin and vimentin protein expression were determined by Western blotting. All samples were total unified total proteins by β‐actin. The pictures were taken under a microscope before protein extraction

Epithelial‐mesenchymal transition (EMT) has emerged as a critical regulator of metastasis in some cancers by conferring an invasive phenotype. We knock down UPP1(Si‐UPP1‐3) find the decreased N‐cadherin, vimentin by Western blot assays, which plays critical roles in EMT pathways. And UPP1 could continue to restrain EMT after the deal with 5‐FU (IC50, 28μM) for 24 hours (Figure 7).

## 4 DISCUSSION

The thyroid gland is a crucial endocrine organ in human, and thyroid hormones play important roles in organ development and homeostasis.^16^ Thyroid hormones regulate skeletal, and brain development in babies and toddlers, control acquisition of peak bone mass and influence vascular remodel in adults.^17^, ^18^, ^19^ Thyroid nodules are extraordinarily prevalent in the coastal areas, giving rise to the fashion of thyroid gland check‐up. Then, we see the continual increases in the incidence of thyroid cancer worldwide.^1^, ^20^, ^21^ With the development of economy and technology, people's request for improving the life quality and healthy standard becomes higher. Surgery is the main treatment for papillary thyroid cancer, but the surgical management of papillary thyroid carcinoma remains contentious.^22^ The extent of thyroid resection has been the nub of this debate. Some experts put forward a thyroid lobectomy could be a conservative approach to treat low‐risk PTC, and the total thyroidectomy remains the standard of care for high‐risk PTC. The occult cancerous foci have been incriminated for higher risk of recurrent disease and its adverse sequelae. The tumorigenesis is mainly decided by genomic variation. Different molecular biomarkers could predict the clinical progress and metastasis of PTC. Finding new molecular biomarkers assess the degree of PTC risk is indispensable.

Uridine phosphorylase 1 encodes uridine phosphorylase catalyses (Upase) and plays an essential anabolic enzyme pyrimidine salvage pathway regulation. Pyrimidines are structural components of key molecules that participate in cellular metabolism. The metabolism of Pyrimidine covers substantially all enzymes of the synthesis, degradation, salvage, interconversion and transport in the cell.^23^ Upase was the first enzyme of the pyrimidine degradative pathway. The lack of Upase would lead to the accumulation of uridine that has been reported a crucial function to reduce 5‐FU toxicity in normal tissues.^9^ BAU, an inhibitor of UPase, is able to protect normal tissues from 5‐Fu host toxicity.^24^, ^25^, ^26^ Also, Upase was discovered to be a higher enzymatic activity in most human tumours. The result of examining the expression of UPase in 72 patients with oral squamous cell carcinoma (SCC) suggests that UPase expression levels were correlated with lymph node metastasis and indicates higher levels of UPase could be a symbol of metastatic phenotype.^11^ This gives us a hint that UPP1 could be a new potential molecular marker in oncogenesis.

In this study, we reports UPP1 was significantly upregulated in thyroid cancer tissues compared with normal thyroid tissues in TCGA dates (Figure 1A) and our verified dates (Figure 1B). Analysed the date of patients with thyroid cancer from TCGA, tumours were significantly smaller, and tumour capsule invasion was seen less frequently in patients with UPP1 low‐expression group. Patients with UPP1 higher expression had more advanced stage (TNM stage III/IV) tumours than the lower group. In addition, the logistic analysis indicated that up‐expression of UPP1 is an independent factor for LNM in PTC. Moreover, we demonstrated that cell proliferation, migration and invasion were significantly inhibited when UPP1 expression was down‐regulated in thyroid cancer cell lines. This seems to suggest that the UPP1 gene is a valuable biomarker for thyroid cancer.

5‐fluorouracil (5‐FU) has been widely used to be a chemotherapeutic agent in the treatment of various cancers across the world.^27^, ^28^ However, 5‐FU–based therapeutic has limited clinical treatment because of dose‐limiting cytotoxicity.^29^ BAU, an inhibitor of UPase, has been reported could reduce 5‐FU toxicity. However, we found the high expression of UPP1 in thyroid cancer. Does that mean the thyroid cancer with lower expression of UPP1 would lower sensitive to 5‐FU? We further tested the impact of UPP1 silencing on 5‐FU chemo sensitivity in thyroid cancer cell lines. Our results demonstrated that silencing of UPP1 would not reduce the inhibition of N‐cadherin and vimentin by 5‐FU. Results of our study shared down‐regulated UPP1 expression in thyroid cancer cell line can inhibit cell metastasis by EMT and do not affect the sensitivity to 5‐FU by EMT.

Our study still contains certain limitations. Although we discovered the potential diagnostic value of UPP1 in thyroid cancer and related EMT, the specific mechanisms of UPP1 in thyroid cancer remain to be further investigated.

## CONFLICT OF INTEREST

The authors declare that they have no conflict of interest.

## AUTHOR CONTRIBUTIONS

Adheesh Bhandari and Yaoyao Guan wrote the manuscript. Yaoyao Guan and Adheesh Bhandari did the main experiments, collected and analysed the raw data. Ouchen Wang helped to revise the article. Ouchen Wang and Xiaohua Zhang designed the whole work.

## CONSENT FOR PUBLICATION

Written informed consent was issued by the patients for the publication of this research and accompanying images. A copy of the written consent is ready for review by the Editor in Chief of this journal.

## ETHICAL APPROVAL AND CONSENT TO PARTICIPATE

Ethical approval for this study was obtained from the Ethics Committee of the First Affiliated Hospital of Wenzhou Medical University.

## Data Availability

The data sets supporting the conclusions of this study are included in this article and its, additional images. Raw data are available on the main electronic data storage system of First Affiliated Hospital of Wenzhou Medical University and access can be provided upon request to the authors.
